# High salt-induced PSI-supercomplex is associated with high CEF and attenuation of state transitions

**DOI:** 10.1007/s11120-023-01032-y

**Published:** 2023-06-22

**Authors:** Isha Kalra, Xin Wang, Ru Zhang, Rachael Morgan-Kiss

**Affiliations:** 1 Department of Microbiology, Miami University, Oxford, OH 45056 USA; 2 Donald Danforth Plant Science Center, St. Louis, MO 63132 USA; 3 Present Address: Department of Biology, University of Southern California, Los Angeles, CA 90089 USA

**Keywords:** Acclimation, Antarctica, *Chlamydomonas*, Cyclic electron flow, PSI-supercomplex, Salinity, State transitions

## Abstract

While PSI-driven cyclic electron flow (CEF) and assembly of thylakoid supercomplexes have been described in model organisms like *Chlamydomonas reinhardtii*, open questions remain regarding their contributions to survival under long-term stress. The Antarctic halophyte, *C. priscuii* UWO241 (UWO241), possesses constitutive high CEF rates and a stable PSI-supercomplex as a consequence of adaptation to permanent low temperatures and high salinity. To understand whether CEF represents a broader acclimation strategy to short- and long-term stress, we compared high salt acclimation between the halotolerant UWO241, the salt-sensitive model, *C. reinhardtii*, and a moderately halotolerant Antarctic green alga, *C.* sp. ICE-MDV (ICE-MDV). CEF was activated under high salt and associated with increased non-photochemical quenching in all three *Chlamydomonas* species. Furthermore, high salt-acclimated cells of either strain formed a PSI-supercomplex, while state transition capacity was attenuated. How the CEF-associated PSI-supercomplex interferes with state transition response is not yet known. We present a model for interaction between PSI-supercomplex formation, state transitions, and the important role of CEF for survival during long-term exposure to high salt.

**Supplementary Information:**

The online version contains supplementary material available at 10.1007/s11120-023-01032-y.

## Introduction

Exposure to abiotic stress leads to disruptions in cell homeostasis, including over-reduction of the photosynthetic electron transport chain (PETC), resulting in oxidative stress and photoinhibition. Photosynthetic organisms have evolved multiple strategies to avoid redox imbalance to support robust growth and photosynthesis (Hüner et al. 2012). In their natural environments, organisms regularly encounter a myriad of environmental stresses which may last for a few minutes (short term or transient) or persist for days to years (long term to permanent) (Hüner et al. 2022; Kono and Terashima 2014). Climate change is exacerbating the unpredictability in the intensity, duration, and frequency of numerous environmental stresses (e.g., extreme storms, flooding, cold snaps, drought, heatwaves, atmospheric CO_2_ levels, soil salinization) (Deryng et al. 2014; Hummel et al. 2018; Hussain et al. 2019). Photosynthetic organisms respond to environmental perturbations either through short-term acclimatory responses such as state transitions or long-term reorganization of the photosynthetic apparatus and/or shifts in downstream carbon metabolism.

The alternative electron transport pathway, PSI-driven cyclic electron flow (CEF) contributes to stress acclimation by supporting photoprotection and/or supplying extra ATP (Kramer and Evans 2011; Suorsa 2015). Research to date on CEF has intensively focused on plants and algae exposed to short-term stress, where activation of maximal CEF rates is transient (Fan et al. 2016; Herbert et al. 1995; Huang et al. 2017; Nawrocki et al. 2019; Saroussi et al. 2016). CEF activation has also been reported under long-term stress, including high salinity, drought, cold temperatures, and nutrient deficiency (He et al. 2015; Huang et al. 2012, 2016, 2017; Suorsa 2015), suggesting that there is a broader role for CEF in both short- and long-term stress acclimation.

While details on mechanism(s) of CEF initiation are still debated, remodeling of PSI and formation of PSI-supercomplexes of varying composition has been associated with high CEF (Alric 2010; Iwai et al. 2010; Minagawa 2016; Steinbeck et al. 2018; Terashima et al. 2012). One of the best characterized PSI-supercomplexes is the PSI–LHCI–LHCII supercomplex formed during short-term photoacclimation (i.e., during a state 1–state 2 transition). In *C. reinhardtii*, assembly of this transitory complex is mainly dependent upon reversible phosphorylation of Type I (LHCB3/4/8) and Type IV (LHCBM1) LHCII proteins (Huang et al. 2021; Iwai et al. 2010; Minagawa 2016). Additional components, including proteins of cytochrome b_6_f (cyt b_6_f), ferredoxin NADP reductase (FNR), proton gradient-like protein 1 (PGRL1), calcium-sensing protein (CAS), and other minor proteins have been reported in various PSI-supercomplexes from *C. reinhardtii* under the state 2 condition (Huang et al. 2021; Steinbeck et al. 2018; Takahashi et al. 2016; Terashima et al. 2012). Recently, Steinbeck et al. (2018) reported two structurally distinct supercomplexes in *C. reinhardtii*, a PSI–LHCI–LHCII and a PSI–LHCI–Cyt *b*_*6*_*f* supercomplex, and predicted distinct roles for each, with the former involved in redistribution of absorbed energy, while the latter complex was attributed to promoting high CEF. Last, several other PSI-supercomplexes have been isolated from other organisms, including the halophile *Dunaliella salina* (Varsano et al. 2006) and a desert alga, *Chlorella ohadi* (Caspy et al. 2021). These studies indicate a connection between CEF and PSI plasticity under different environmental conditions; however, there is a lack of an integrated theme between PSI structural variation and the functional consequences for long-term stress survival.

The Antarctic *Chlamydomonas priscuii* UWO241 (UWO241 henceforth) was isolated from the deep photic zone of an Antarctic lake (Lake Bonney, McMurdo Dry Valleys) (Morgan et al. 1998; Neale and Priscu 1990, 1995; Stahl-Rommel et al. 2021). It is a psychrophilic, halotolerant alga and survives under temperatures near the freezing point of water (− 2 to 5 °C) and high salt (up to 1 M NaCl) (Morgan et al. 1998; Morgan-Kiss et al. 2006). Isolated in a stratified water column under a year-round ice cover, UWO241 has evolved in a stable, extreme habitat for at least thousand years (Poreda et al. 2004). More than two decades of study of UWO241 have described its novel adaptive strategies, in particular, remodeling of the photosynthetic apparatus (reviewed in Cvetkovska et al. 2017), a consequence of which is complete loss of state transitions and a reliance on energy spill-over (Morgan-Kiss et al. 2002a, b; Szyszka-Mroz et al. 2019). Under low-temperature and high-salt conditions, UWO241 exhibits high CEF which is associated with assembly of a stable PSI-supercomplex (Morgan-Kiss et al. 2002b; Szyszka-Mroz et al. 2015; Kalra et al. 2020). Proteins from PSI core, Cyt b_6_f, and chloroplastic ATP synthase were detected in the UWO241 supercomplex, as well as CEF-associated CAS and PGRL1, and two novel phosphor proteins, FtsH, and a PsbP (Szyszka-Mroz et al. 2015; Kalra et al. 2020). Thus, high CEF supported by a stable PSI-supercomplex are a major strategy for long-term stress survival in UWO241, providing both extra ATP production and constitutive NPQ capacity (Kalra et al. 2020).

With intensified agricultural practices, increased salinity stress is frequently encountered by crops (Morton et al. 2019; Welle and Mauter 2017). Concern for impact of increasing salinization on phytoplankton communities in freshwater and coastal ecosystems is also growing (Melles et al. 2023). Plants and algae possess a number of adaptive responses to high salt (Mahajan and Tuteja 2005). Survival in high salt requires additional ATP (e.g.,: ion homeostasis, production of osmolytes) (Goyal 2007; He et al. 2015); therefore, plants exposed to high salt are under a condition of unbalanced energy production. CEF constitutes a major pathway by which photosynthetic organisms fine tune the ATP/NADPH ratio based on downstream energetic demands (Suorsa 2015) and could play a significant role in providing extra ATP under salt stress. A significant body of research has focused on salinity stress response in model algal species with minimal salt tolerance, such as *C. reinhardtii*. Moreover, many of these previous studies were performed under mixotrophic growth conditions whereby photosynthesis is nonessential (Neelam and Subramanyam 2013; Sudhir and Murthy 2004; Wang et al. 2018; Perrineau et al. 2014; Sithtisarn et al. 2017; Heifetz et al. 2000).

There is a growing interest to include experiments on a repertoire of ‘wild’ *Chlamydomonas* spp. which possess broader tolerances to environmental disturbances (Grossman 2021). UWO241 is one of a handful of photopsychrophiles which have been extensively used to understand survival of photosynthetic organisms under persistent and extreme environmental stress. Recently, a second photopsychrophile, named *Chlamydomonas* sp. ICE-MDV (ICE-MDV henceforth) was isolated from Lake Bonney, where it dominates the algal communities of the freshwater surface layers (Li and Morgan-Kiss 2019; Li et al. 2016). In contrast with UWO241, ICE-MDV has adapted to relatively low salt conditions which appears to have resulted in significant distinctions in the photochemical apparatus between the two psychrophiles (Cook et al. 2019). In this study, we investigated the impact of variable salinity tolerance across three *Chlamydomonas* spp. and asked whether CEF and PSI-supercomplexes are a common strategy to fine tune redox status of PETC under long-term salinity stress. In addition, we hypothesized that high-salt-associated PSI-supercomplexes may disrupt state transition capacity.

## Methods

### Culture conditions, growth physiology

Three different *Chlamydomonas* species were used in this study: *C*. *priscuii* UWO241 (UWO241 hereafter; strain CCMP1619), *C*. sp. ICE-MDV (ICE-MDV hereafter), and the model *C. reinhardtii* (strain UTEX 90). All three species were first grown in Bold’s Basal Media (BBM, 0.43 mM NaCl) (Low salt, LS). UWO241 and ICE-MDV cultures were grown under a temperature/irradiance regime of 8 °C/50 μmol photons m^−2^ s^−1^ which closely matches native temperature/light conditions and matches previous growth conditions (Cook et al. 2019; Morgan et al. 1998). *C. reinhardtii* UTEX 90 was grown in BBM (LS) at 20 °C/100 μmol photons m^−2^ s^−1^. All cultures were grown in 250 ml glass pyrex tubes in temperature regulated aquaria under a 24 h light cycle and were continuously aerated with sterile air supplied by aquarium pumps (Morgan-Kiss et al. 2002b).

For the salinity tolerance experiments, cultures were grown in increasing concentration of NaCl supplemented BBM (0.43–200 mM NaCl for *C. reinhardtii*, 0.43–700 mM NaCl for ICE-MDV and 0.43–1000 mM for UWO241). Growth was monitored by optical density at a wavelength of 750 nm. Growth rates were calculated using natural log transformation of the optical density values during the exponential phase. Three biological replicates were performed.

For salinity stress acclimation, cultures were grown in maximum tolerated salinity levels and sub-cultured after reaching log-phase, at least 2–3 times. All subsequent experiments were conducted on low-salinity (LS) and high-salinity acclimated (HS) log-phase cultures.

### Room-temperature PSII chlorophyll fluorescence measurements

Photosynthetic measurements were conducted using room-temperature PSII chlorophyll fluorescence through Dual PAM-100 instrument (Walz, Germany). Briefly, 2 ml of exponentially grown cultures were dark adapted using far-red light for 2 min prior to the measurement. For steady-state analysis, we used induction curves to measure maximum capacity of photosynthesis (*F*_V_/*F*_M_), photosynthetic yield (YPSII), non-photochemical quenching (YNPQ), and photochemical yield (qP) with actinic light set at growth light intensities for each species. Light curves in Dual-PAM were also conducted to measure the change in capacity of NPQ with increasing light levels.

### State transition induction

State transition experiments were conducted on both low- and high-salinity acclimated cultures. Briefly, cultures were harvested in the mid-log-phase and induced in either state 1 or state 2 through addition of chemical inhibitors as described before (Iwai et al. 2010). For state 1 induction, mid-log-phase cells were incubated in 10 µM DCMU to completely oxidize the PQ pool prior to measurement. For state 2 induction, cells were incubated in 5 µM FCCP for 20 min. State transition response was measured through either 77 K fluorescence emission or PSII fluorescence as described below.

### Low-temperature (77 K) fluorescence spectra

Low-temperature fluorescence spectra were conducted as previously described (Morgan et al. 1998). Briefly, log-phase cultures (~ 5 μg/ml chlorophyll) or diluted isolated Chl-protein complexes (5–10 μg/ml chlorophyll) were dark adapted for 10 min and flash frozen in liquid N_2_ before the measurement. Frozen samples were exposed to excitation wavelength of 436 nm with slit widths of 8 nm for whole cells and 5 nm for isolated complexes in a continuously cooled environment (Morgan-Kiss et al. 2008). For each sample, at least three replicates of emission spectra were measured.

### PSII fluorescence state transition measurement

Room-temperature PSII fluorescence measurements were conducted on cultures induced in state 1 or state 2 as described above. Preliminary analysis was done to identify PSII saturating actinic light intensity and 200 μmol photons m^−2^ s^−1^ was chosen for the subsequent measurements. Log-phase exponentially growing cultures (2 ml) were used for the measurement. Briefly, measuring light was switched on in the dark and minimal PSII fluorescence (*F*_O_) was measured. Subsequently, cultures were exposed to 200 μmol photons m^−2^ s^−1^ of actinic red light (*λ*_max_ = 620 nm, 10 Wm^−2^, Scott filter RG 715) to measure maximum fluorescence (*F*_M_). Percent state transition capacity was calculated using *F*_M_ values measured under state 1 and state 2 using the formula (*F*_M_^ST1^ − *F*_M_^ST2^)/*F*_M_^ST1^% as described before (Girolomoni et al. 2017), where *F*_M_^ST1^ and *F*_M_^ST2^ are the maximal PSII fluorescence under state 1 and 2, respectively.

### P700 oxidation–reduction kinetics

Actinic red light-induced photooxidation–reduction of P700 was used to determine rates of CEF as previously described (Morgan-Kiss et al. 2002b; Alric et al. 2010). Exponential phase cultures (~ 25 μg Chl) were dark adapted for 10 min in the presence of DCMU to block electrons from PSII. Dark-adapted cultures were then filtered onto 25 mm GF/C filters (Whatman) and measured on the Dual-PAM-100 instrument using the leaf attachment. Absorbance changes at 820 nm were used to calculate proportion of photo-oxidizable P700, expressed as the parameter ∆*A*_820_/*A*_820_. To start the measurement, the signal was balanced and measuring light was switched on. First, P700 was oxidized (P700^+^) by switching on the actinic red light (AL, *λ*_max_ = 620 nm, 10 Wm^−2^, Scott filter RG 715). Subsequently, AL was switched off to re-reduce P700^+^ after steady-state oxidation was reached. The half-time for the reduction of P700^+^ to P700 (*t*_½_^red^) was calculated as an estimate of relative rates of PSI-driven CEF (Ivanov et al. 1998). The re-reduction half-time for P700 was calculated using GraphPad Prism™ software.

### In vivo spectroscopy measurements

Dark Interval Relaxation Kinetics (DIRK) of electrochromic shift (ECS) were performed on the Kramer Lab IDEA spectrophotometer (Sacksteder and Kramer 2000; Zhang et al. 2009) to evaluate proton fluxes across thylakoid membrane. Simultaneously, saturation-pulse chlorophyll fluorescence was also measured to estimate the PSII operating efficiency ($\phi$PSII). Measurements were performed as described before (Kalra et al. 2020). Briefly, 2.5 ml (~ 12 μg/ml chlorophyll) of exponential phase *C. reinhardtii* culture was incubated in the presence of bicarbonate under dark condition for 10 min followed by far-red exposure for 10 min, to fully oxidize the plastoquinone pool. Cells were incubated in increasing actinic red light for 5 min, and ECS and chlorophyll fluorescence were measured after each light incubation. The PSII operating efficiency was calculated using the formula: (*F*_M′_ − *F*_S_)/*F*_M_, and linear electron flow (LEF) was calculated using the equation *A* × (*fraction*_*PSII*_) × *I* × $\phi$*PSII* (Baker 2008), where *A* is absorptivity of the sample (assumed to be 0.84), *fraction*_*PSII*_ is the fraction of light absorbed by PSII stimulating photosynthesis, *I* is the irradiance used and $\phi$*PSII* is the operating efficiency of PSII as calculated above. In the above equation, *fraction*_*PSII*_ was calculated using the 77 K fluorescence spectra and the fraction was 0.56 and 0.579 for low and high salinity conditions, respectively. Proton motif force (pmf) was estimated by using the total amplitude of ECS signal (ECS_t_), and the total proton conductivity (g_H_^+^) of thylakoid membranes was estimated through the inverse of lifetime of rapid decay of ECS signal ($\tau$_ECS_) during the DIRK measurements (Baker et al. 2007).

### Thylakoid isolation, SDS-PAGE, and immunoblotting

Thylakoid membranes were isolated according to previously described protocol (Morgan et al. 1998). Briefly, log-phase cells were harvested using centrifugation (2500×*g* at 4 °C for 5 min) and resuspended in the cold grinding buffer (0.3 M sorbitol, 10 mM NaCl, 5 mM MgCl_2_, 1 mM benzamidine, and 1 mM amino-caproid acid). The resuspension was passed through a chilled French press at 10,000 lb/in^2^ two times followed by centrifugation at 23,700×*g* for 30 min to collect thylakoid membranes. The membranes were then washed to remove any impurities using wash buffer (50 mM Tricine-NaOH [pH 7.8], 10 mM NaCl, 5 mM MgCl_2_) and the pure thylakoid membranes were collected (13,300×*g* at 4 °C for 20 min). Membranes were resuspended in storage buffer and kept at − 80 °C until further use.

SDS-PAGE and immunoblotting were performed using 12% Urea-SDS gel (Laemmli 1970) and as previously described (Kalra et al. 2020). Phosphorylated threonine sites were probed using a primary antibody against P-Thr (Catalog # MA5-27976, Thermo Fisher) at 1:500 dilution followed by exposure to protein A conjugated to horseradish peroxidase. Protein blots were detected using ECL Select™ Western Blotting Detection Reagent (Amersham).

### Supercomplex isolation

Sucrose density gradient centrifugation was used to isolate supercomplexes from exponentially grown cultures as previously described (Kalra et al. 2020; Szyszka-Mroz et al. 2015). All steps were performed in darkness and on ice. All buffers contained phosphatase (20 mM NaF) and protease (1 mM Pefabloc SC) inhibitors. Protein complexes were extracted using a 21-gage needle for further analysis.

### Sample preparation for proteomics

For identifying protein components in the supercomplex, the complex was harvested and 30 μg of total protein was processed for proteomics following the previously published method by Wang et al. (2016). Samples were digested and cleaned as described before (Kalra et al. 2020).

### Proteomic analyses by liquid chromatography–tandem mass spectrometry (LC–MS/MS)

2 μg of digested peptides were directly loaded onto a capillary C18 column without fractionation and analyzed in a Thermo LTQ Orbitrap XL mass spectrometer. The full mass spectra in the range of 350–1800 *m/z* were recorded with a resolution of 30,000, and the top 12 peaks of each scan were then selected for further fragmentation for MS/MS analysis. The MS/MS raw data were analyzed using the Patternlab for Proteomic tool (Carvalho et al. 2016). Our UWO241 transcriptomics data were used to generate a UWO241 protein database after supplementing with 37 common contaminants. Reversed sequences were also included as a quality control system to restrain false-positive discovery rate to 0.05. *C. reinhardtii* protein database was downloaded from NCBI containing both Swiss-Prot and TrEMBL entries.

## Results

### Salinity tolerance comparison

We monitored growth physiology across salinity gradients to confirm maximum salinity concentration tolerated for each strain. Based on the native habitat of Lake Bonney combined with previous literature on salt tolerance in UWO241 (Pocock et al. 2011) and *C. reinhardtii* (Subramanyam et al. 2010), we selected the following salinity ranges: (i) *C. reinhardtii* was tested at a salinity range of 0.43 mM NaCl (BBM media) to 200 mM NaCl, (ii) ICE-MDV was tested at a salinity range of 0.43 mM NaCl to 700 mM added NaCl, and (iii) UWO241 was tested at a salinity range of 0.43–1000 mM NaCl (Fig. S1). The model mesophile *C. reinhardtii* grew maximally in BBM medium (0.43 mM NaCl, *µ*_max_ of 0.0088 ± 0.0002 h^−1^) and grew logarithmically up to an NaCl concentration of only 50 mM (*µ*_max_ of 0.0071 ± 0.0015 h^−1^). Growth rates of *C. reinhardtii* declined rapidly at and beyond added salinity of 100 mM (Fig. S1). On the other hand, for UWO241, maximum growth rates were observed under the highest salinity tested, 700 mM NaCl (*µ*_max_ of 0.0072 ± 0.0005 h^−1^), 1.1-fold faster relative to BBM-grown cultures of UWO241 (Fig. S1, Table 1). However, UWO241 could not grow at salinity level of 1000 mM NaCl. In contrast with UWO241, ICE-MDV exhibited maximal growth rates in BBM (0.43 mM NaCl, *µ*_max_ of 0.0074 ± 0.00005 h^−1^). Maximum salinity concentration tolerated by ICE-MDV was 500 mM NaCl (*µ*_max_ of 0.0059 ± 0.0013 h^−1^), and the growth rate was 1.2-fold slower compared to UWO241 grown in 700 mM NaCl (Fig. S1). Thus, ICE-MDV possesses significantly higher salt tolerance compared to the model *C. reinhardtii*, but lower than its sister species, UWO241. Based on these results, we chose the following salinity levels for further experiments testing long- and short-term acclimation responses. For low-salinity (LS) experiments, all strains were grown in BBM (0.43 mM NaCl). For high salinity (HS), we used BBM supplemented with, (i) 50 mM NaCl for *C. reinhardtii*, (ii) 500 mM NaCl for ICE-MDV, and (iii) 700 mM NaCl for UWO241*.* Photosynthetic and physiological measurements indicated that all three strains maintained a comparable high measures of photochemical activity across all salinity conditions (Table 1).

**Table 1:** Physiological characterization of low salt (LS) and high salt (HS)-acclimated *Chlamydomonas* species

	*C. reinhardtii*		ICE-MDV		UWO241	
	LS	HS	LS	HS	LS	HS
Doubling time (hr)	30.34 ± 2.24	32.85 ± 6.62	48.53 ± 0.15	50.48 ± 7.09	47.66 ± 3.40	29.64 ± 2.88
F_V_/F_M_	0.720 ± 0.011	0.697 ± 0.019	0.694 ± 0.009	0.669 ± 0.004	0.640 ± 0.008	0.585 ± 0.007
YPSII	0.621 ± 0.006	0.555 ± 0.068	0.536 ± 0.019	0.518 ± 0.007	0.463 ± 0.001	0.524 ± 0.029
qP	0.909 ± 0.060	0.829 ± 0.056	0.733 ± 0.026	0.798 ± 0.025	0.719 ± 0.089	0.766 ± 0.088
NPQ	0.184 ± 0.028	0.230 ± 0.037	0.171 ± 0.017	0.223 ± 0.007	0.256 ± 0.026	0.307 ± 0.043
Chlorophyll (μg/ml)	4.38 ± 0.43	3.92 ± 0.54	3.98 ± 0.07	3.14 ± 0.12	3.83 ± 0.18	2.21 ± 0.12

Doubling time, photosynthetic parameters, and extracted chlorophyll content are shown for all three species (UWO241, ICE-MDV, and *C. reinhardtii*) under the two salinity conditions, low salt (LS) and high salt (HS) (*n* = 3 ± SD)

*F*_*v*_*/F*_*M*_ maximal photosynthetic capacity, *YPSII* PSII yield, *NPQ* non-photochemical quenching, *qP* photochemical efficiency

### Impact of salt stress on CEF and PSI activity

**Fig. 1:**
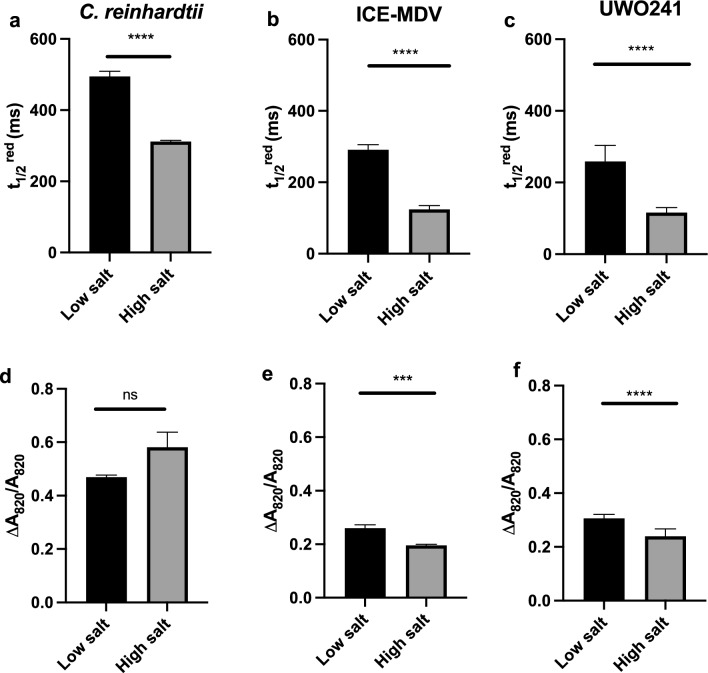
P700 oxidation/reduction analysis on the three *Chlamydomonas* spp. under low and high salinity. Top panel: Re-reduction half-time (*t*_1/2_^red^) was calculated under low (black) and high salinity (gray) for all three strains: *C. reinhardtii* (**a**), ICE-MDV (**b**), UWO241 (**c**). Bottom panel: The proportion of photo-oxidizable P700 is shown as change in absorbance at 820 nm (∆*A*_820_/*A*_820_) for all three strains under low and high salinity: *C. reinhardtii* (**d**), ICE-MDV (**e**), UWO241 (**f**). Actinic red light was used with DCMU to inhibit electron flow from PSII [*n* = 9, $\pm$ SD, ns (not significant, *p* > 0.05), ** (*p* < 0.01), *** (*p* < 0.005), **** (*p* < 0.001)]

P700 oxidation/reduction kinetics were measured in log-phase cultures to monitor changes in P700 photooxidation activity and PSI-mediated CEF (Figs. 1, S2) before and after salinity stress acclimation. In accordance with our hypothesis, we observed that the model *C. reinhardtii* displayed 1.5-fold faster re-reduction half-time after acclimation to salinity stress (*t*_½_^red^ = 311 ± 10 ms; Fig. 1a) when compared to LS cultures (*t*_½_^red^ = 495 ± 43 ms) (Fig. 1a) which matched the salinity response of the psychrophiles. On the other hand, *C. reinhardtii* exhibited a slower *t*_½_^red^ in LS media (*t*_½_^red^ = 495 ± 43 ms) which was around 1.6–1.9-fold slower than LS-grown ICE-MDV and UWO241 (Fig. 1a). Similar to the response of *C. reinhardtii*, ICE-MDV also responded to HS acclimation by a 2.3-fold faster *t*_½_^red^ (*t*_½_^red^ = 124 ± 32 ms). However, the psychrophile ICE-MDV had a 1.6-fold faster *t*_1/2_^red^ compared to *C. reinhardtii* under low-salinity condition (*t*_1/2_^red^ = 291 ± 42 ms) (Fig. 1b). UWO241 showed the fastest *t*_1/2_^red^ (*t*_½_^red^ = 259 ± 45 ms) among the three strains under low salinity, which further increased 1.6-fold (*t*_½_^red^ = 162 ± 14 ms) after acclimation to HS in agreement with previous literature (Kalra et al. 2020; Szyszka-Mroz et al. 2015). Overall, the psychrophiles displayed faster *t*_1/2_^red^ in both LS and HS conditions compared to *C. reinhardtii*.

In addition to CEF rates, we also measured change in P_700_ absorbance (∆*A*_820_/*A*_820_) after AL illumination which reflects the redox state of P700. Compared to the model *C. reinhardtii* (Fig. 1d), both psychrophiles ICE-MDV and UWO241 (Fig. 1e, f) displayed lower ∆*A*_820_/*A*_820_ (1.5- and 1.7-fold lower, respectively) indicating a lower capacity for PSI oxidation. Moreover, while *C. reinhardtii* showed no significant change (Fig. 1d), both the psychrophiles showed further significant reduction ∆*A*_820_/*A*_820_ after salinity acclimation (Fig. 1e, f).

### ECS in *C. reinhardtii* in response to salinity

**Fig. 2:**
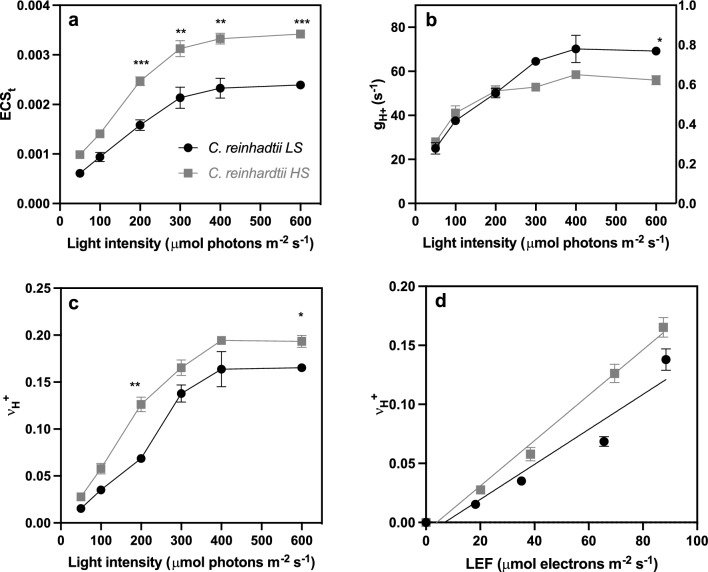
Electrochomic shift (ECS) measurements for *C. reinhardtii* after low and high salinity acclimation. **a** Total proton motive force (pmf) is shown as total change in the ECS signal (ECS_t_) for low (LS) and high (HS) salt-acclimated cultures under increasing light intensities. **b** ATP synthase conductance (g_H_^+^). **c** Total proton flux (v_H_^+^) and **d** Change in total proton flux as a function of linear electron flow (LEF) [*n* = 3, $\pm$ SD,* (*p* < 0.05), ** (*p* < 0.01), *** (*p* < 0.005), **** (*p* < 0.001)]

To complement the P700 results, we measured electrochromic shift (ECS) kinetics in *C. reinhardtii* LS and HS cultures (Fig. 2). ECS kinetics were previously also measured in UWO241 (Kalra et al. 2020). ECS estimates light-dependent photosynthesis-driven transthylakoid proton flux using IDEA spectrophotometer (Baker et al. 2007) (Fig. 2). We used dark interval relaxation kinetics (DIRK) to observe the shifts in the membrane potential (electrochromic signal at 520 nm) (Kramer et al. 2003). The proton motive force (pmf) was estimated from the total ECS signal (ECS_t_) in *C. reinhardtii* under both low and high salinity conditions (Fig. 2a). We detected higher pmf in HS-acclimated *C. reinhardtii* in all light intensities, which was significantly higher under light intensities of 200 µmol photons m^−2^ s^−1^ and above, compared to low salt conditions (~ 1.6-fold). This increase in pmf can be attributed to either decrease in ATP synthase activity or proton efflux, or an increase in proton flux through LEF or CEF. To identify the processes contributing to increased pmf in HS cultures, we measured the transthylakoid proton conductivity (g_H_^+^) and flux (v_H_^+^) through ATP synthase (Carrillo et al. 2016; Kanazawa and Kramer 2002; Livingston et al. 2010) (Fig. 2b, c). Proton conductivity or permeability (g_H_^+^) is estimated from inverse of lifetime of rapid decay of the ECS signal ($\tau$_ECS_) and is dependent on ATP synthase activity (Baker et al. 2007). Both LS and HS cultures showed similar conductivity at lower light intensities; however, at 300 µmol photons m^−2^ s^−1^ and above, the conductivity in HS cultures decreased (~ 1.2-fold) compared to LS cultures (Fig. 2b). On the other hand, the proton flux in HS cultures was consistently higher (1.2 to 2.0-fold) compared to LS cultures, indicating higher ATP synthesis in HS condition (Fig. 2c). To dissect whether LEF or CEF is contributing to increased proton flux in HS cultures, we plotted v_H_^+^ against LEF. The slope of this curve can inform us about the relative contribution of each pathway towards proton flux (Fig. 2d). We observed that the slope of the HS cultures was 1.2-fold higher than LS cultures, indicating that CEF significantly contributes towards increased proton flux in *C. reinhardtii*-HS cultures, which tightly corroborates with our P700 findings. Similar results were also observed for UWO241 cultures adapted to high salinity in our previous study (Kalra et al. 2020), further emphasizing the importance and ubiquity of CEF in HS acclimation in both non-halophyte and halophyte *Chlamydomonas* species.

### Impact of salinity on 77 K fluorescence emission spectra and state transitions

**Fig. 3:**
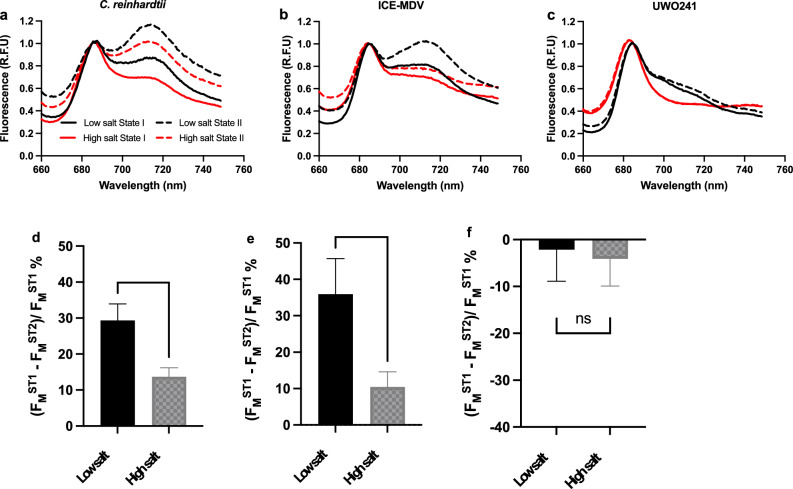
State transition tests after acclimation to low and high salinity in *Chlamydomonas* species. Top panel: Low-temperature (77 K) fluorescence spectra of the three *Chlamydomonas* spp. under state I and state II conditions after low and high salinity acclimation. Fluorescence values are shown as relative fluorescence units (R.F.U) for each strain: *C. reinhardtii* (**a**); ICE-MDV (**b**); UWO241 (**c**). Low salinity—Black, High salinity—Red. State I—Closed line, State II—dotted line. Bottom panel: Maximal capacity for switching LHCII antenna during State transition induction calculated using room-temperature PSII maximum fluorescence (*F*_M_) as described before (Girolomoni et al. 2017) for each strain: *C. reinhardtii* (**d**); ICE-MDV (**e**); UWO241 (**f**). *ST1* state 1, *ST2* state 2 [*n* = 4–6; $\pm$ SD; ns (not significant, *p* > 0.05), ** (*p* < 0.01), *** (*p* < 0.005)]

Energy partitioning between PSII and PSI was measured by Chl a fluorescence emission at 77 K (Fig. 3). Under LS, *C. reinhardtii* exhibited a typical fluorescence emission spectrum with well-defined peaks for PSII and PSI, while acclimation to HS resulted in a 1.2-fold decrease in PSI fluorescence yield (Fig. 3a). LS-grown ICE-MDV exhibited 1.1-fold lower levels of PSI fluorescence relative to *C. reinhardtii*. HS-grown ICE-MDV also exhibited a 1.2-fold reduction PSI fluorescence emission relative to LS-grown cells (Fig. 3b). In contrast with both *C. reinhardtii* and ICE-MDV and in agreement with previous reports (Morgan-Kiss et al. 2002b; Kalra et al. 2020), UWO241 cells exhibited minimal PSI fluorescence regardless of the salinity growth regime (Fig. 3c).

We measured the impact of salt acclimation on state transition capacity by comparing 77 K fluorescence emission under state 1 versus state 2 conditions (Fig. 3). First, *C. reinhardtii* exhibited a comparable capacity for state transition under either LS or HS growth conditions, as indicated by a ~ 1.4-fold increase in PSI fluorescence under state 2 conditions (Fig. 3a). ICE-MDV also showed the capacity for state transition as LS-grown ICE-MDV exhibited a 1.25-fold increase in PSI fluorescence under state 2 treatment (Fig. 3b). However, in contrast with *C. reinhardtii*, HS-grown ICE-MDV exhibited relatively smaller changes in PSI fluorescence (1.1-fold) under state 2. Last, UWO241 cells grown in either salt regime exhibited no change in their 77 K fluorescence emission spectra (Fig. 3c), confirming previous reports that it is a natural state transition mutant (Morgan-Kiss et al. 2002b).

Apart from measuring PSI fluorescence dynamics, state transitions can also be detected via reduction in maximum fluorescence of PSII (*F*_M_) at room temperature in state 2 versus state 1 conditions (Girolomoni et al. 2017). The migration of phosphorylated LHCII antenna to PSI during state 2 leads to decrease in the maximal fluorescence of PSII (*F*_M_), which can be measured. Here, state transition capacity is defined as the percent decrease in *F*_M_ in state 2 vs state 1 condition using the formula (*F*_M_^ST1^ − *F*_M_^ST2^)/*F*_M_^ST1^ × 100%. In agreement with the results of 77 K fluorescence emission, LS-grown cultures of *C. reinhardtii* and ICE-MDV cells both exhibited state transition capacity, as measured by a loss in *F*_M_ in state 2 versus state 1 (29% and 36% loss in *F*_M_, respectively; Fig. 3d, e; Fig. S3a, b). A decrease in state transition capacity would reflect as decrease in migration of LHCII to PSI which can be measured as a decrease in (*F*_M_^ST1^ − *F*_M_^ST2^) in our measurement. We observed that acclimation to HS resulted in a 2.2- and 3.5-fold loss in state transition capacity in *C. reinhardtii* and ICE-MDV, respectively. As seen in previous research and our 77 K data above, UWO241 exhibited minimal changes in *F*_M_ regardless of the salt regime (Fig. 3f, Fig. S3c).

### Interactions between CEF rates and NPQ capacity

**Fig. 4:**
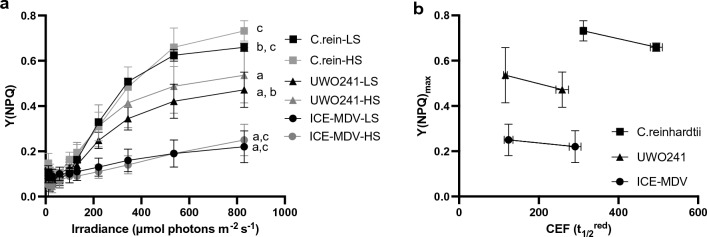
Effect of salinity on non-photochemical quenching (NPQ) capacity and relationship with cyclic electron flow (CEF). **a** NPQ capacity (Y(NPQ)) was measured for the three species during a light curve under low (LS, black) and high salinity (HS, gray) conditions in the three strains *C. reinhardtii* (*C. rein*, square), UWO241 (triangle) and ICE-MDV (circles). Statistically significant differences between UWO241 vs ICE-MDV (a) and *C. reinhardtii* (b) as well as *C. reinhardtii* vs ICE-MDV (c) are shown for the highest light intensity (800 μmol m^−2^ s^−1^) (Welch’s *t*-test, *p* < 0.05). **b** Relationship between maximum NPQ capacity (Y(NPQ)_max_) and CEF (re-reduction half-time, *t*_1/2_^red^) is shown for the two salinity conditions for all species. Faster *t*_1/2_^red^ (lower value) indicates higher CEF

Light-response patterns of NPQ were monitored in all three strains acclimated to LS versus HS using rapid light curves (Fig. 4a). Overall, *C. reinhardtii* exhibited a significantly higher NPQ capacity compared with UWO241 and ICE-MDV under either LS or HS conditions. As previously shown, UWO241 showed higher NPQ capacity under high salinity at every light level. On the other hand, *C. reinhardtii* and ICE-MDV showed higher NPQ capacity under high salinity at the maximum light intensity (830 µmol photons m^−2^ s^−1^). We compared the relationship between maximum NPQ capacity and CEF for the three *Chlamydomonas* species (Fig. 4b). High NPQ was correlated with higher CEF rates in all three species (i.e., faster *t*_1/2_^red^).

### Thylakoid protein phosphorylation

**Fig. 5:**
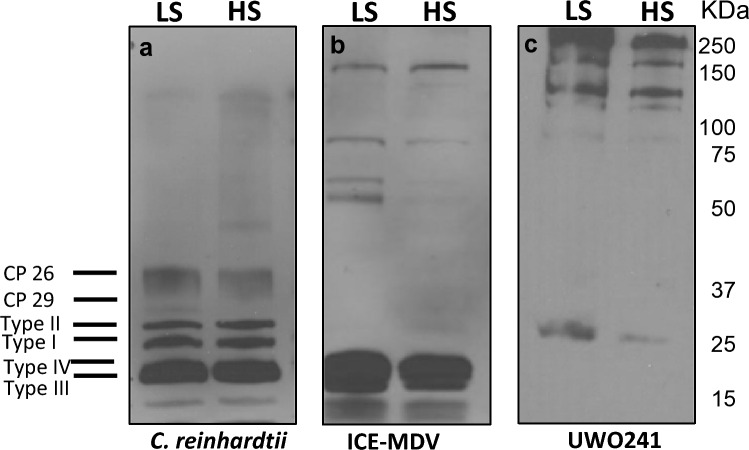
Thylakoid phosphorylation pattern of the three *Chlamydomonas* spp. under low (LS) and high (HS) salinity. Isolated thylakoids were run on 12% SDS-PAGE and probed with phospho-threonine antibody. Three separate PAGE gels and Western blots were conducted for the three species (**a** *C. reinhardtii*, **b** ICE-MDV, **c** UWO241). Molecular weight ladder (kDa) is shown on the right. The different LHCII types are labeled on the left

State transitions are associated with transient phosphorylation of LHCII proteins through STT7 kinase (Lemeille and Rochaix 2010). Since we observed a salinity-associated changes in state transition capacity (Fig. 3), we compared thylakoid phospho-protein profiles of the three strains acclimated to either LS or HS (Fig. 5). When probed with a phospho-threonine antibody, *C. reinhardtii* exhibited typical phosphorylation of several thylakoid proteins, specifically phosphorylated LHCII (type I, II, III and IV); however, HS did not alter the pattern or abundance of phospho-proteins of *C. reinhardtii* (Fig. 5a). ICE-MDV exhibited phosphorylation of some major LHCII proteins (type III and IV) under either LS or HS; however, some phospho-LHCII proteins that were detected in *C. reinhardtii* were not detectable (Fig. 5b). On the other hand, phosphorylation of major LHCII proteins was not detectable in UWO241 grown under either LS or HS conditions (Fig. 5c). As previously reported (Szyszka-Mroz et al. 2015), the phosphoprotein profile of thylakoids of LS- and HS-grown UWO241 exhibited phosphorylation of several high molecular weight proteins (~ 150 and 250 kDa). Interestingly, thylakoids of LS- or HS-grown ICE-MDV also exhibited several high molecular weight phospho-proteins (~ 150 kDa), although, the patterns and abundances were distinct compared with that of UWO241. No higher molecular weight phospho-proteins were detected in *C. reinhardtii.*

### Presence of PSI-supercomplexes in high salinity

**Fig. 6:**
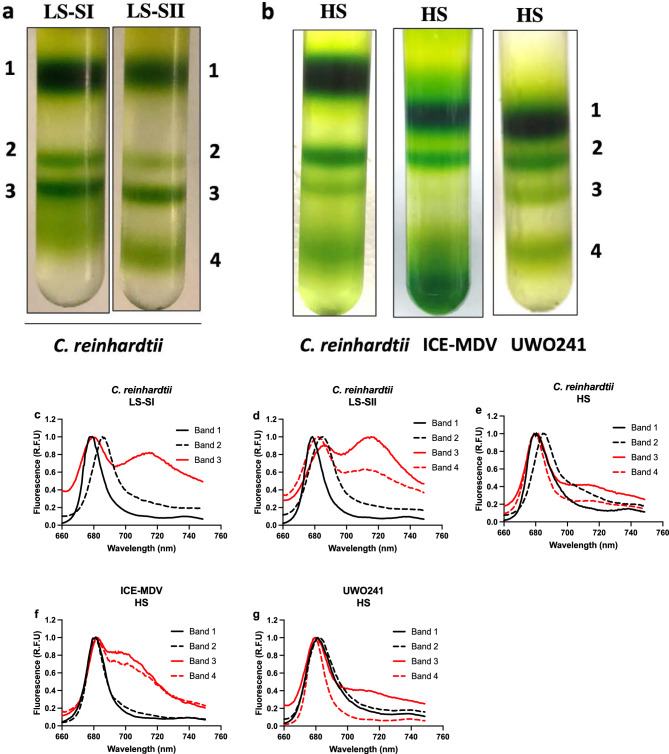
Isolation of supercomplexes from conditions promoting CEF in *Chlamydomonas* species. Top panel: Separation of protein complexes on a sucrose density gradient for **a** model mesophile *C. reinhardtii* during state transitions and **b** model mesophile and the psychrophiles ICE-MDV and UWO241 under high salinity. Bottom panels: 77 K fluorescence spectra for protein complex bands isolated from sucrose density gradient: *C. reinhardtii* under low salt and state 1 (**c**), state 2 (**d**), under high salinity (**e**); ICE-MDV under high salinity (**f**); UWO241 under high salinity (**g**). *LS-SI* low salinity, state 1; *LS-SII* low salinity, state 2; *HS* high salinity. Band 1: LHCII, Band 2: PSII, Band 3: PSI–LHCI, Band 4: Supercomplex

PSI-supercomplexes have been shown to be associated with conditions of high CEF, for example, state transitions in *C. reinhardtii* (Iwai et al. 2010) and long-term salt stress in UWO241 (Szyszka-Mroz et al. 2015; Kalra et al. 2020). Sucrose density gradient centrifugation on solubilized thylakoid membranes was performed and banding patterns of Chl-protein complexes were compared between *C. reinhardtii* exposed to state 1 and state 2 conditions and the three algae grown in HS (Fig. 6). LS-grown *C. reinhardtii* exhibited typical banding patterns, representing (i) trimeric LHCII (band 1), (ii) PSII core (band 2), (iii) PSI–LHCI complex (band 3) under state 1 condition and presence of an additional fourth band (iv) PSI-supercomplex (band 4) associated with the State 2 condition (Fig. 6a). We then compared the banding patterns in sucrose density gradients from thylakoids isolated from all three *Chlamydomonas* species after acclimation to salinity. Thylakoids from HS-acclimated cells of all three strains exhibited a reduction in the relative levels of PSI (Band 3) and the presence of PSI-supercomplex (band 4; Fig. 6b).

Bands from the gradients were collected for 77 K Chl fluorescence analysis (Fig. 6c–g). In *C. reinhardtii* state 1 cells, Band 1 and 2 exhibited major fluorescence peaks at 678 nm (LHCII) and 682 nm (PSII core), respectively, while Band 3 exhibited emission peaks at both 678 nm and 712 nm (PSI) (Fig. 6c). Band 4 from state 2 cells of *C. reinhardtii* exhibited a similar emission spectrum pattern as the PSI band, with a reduction in 712 nm emission (Fig. 6d). The 77 K fluorescence emission spectra of PSI and PSI-supercomplex from thylakoids of HS-acclimated cells exhibited strain-specific distinctions (Fig. 6e–g). HS acclimation in *C. reinhardtii* resulted in a reduction in PSI fluorescence emission at 712 nm in Bands 3 and 4, relative to LS conditions (Fig. 6e). In contrast, HS-acclimated ICE-MDV exhibited a broad shoulder in fluorescence emission between 705 and 712 nm for Bands 3 and 4 (Fig. 6f). In agreement with previous literature (Kalra et al. 2020), HS-acclimated UWO241 exhibited minimal PSI fluorescence for Bands 3 and 4 (Fig. 6g).

### Proteome analysis of high salinity-associated supercomplexes from *C. reinhardtii* and UWO241

**Fig. 7:**
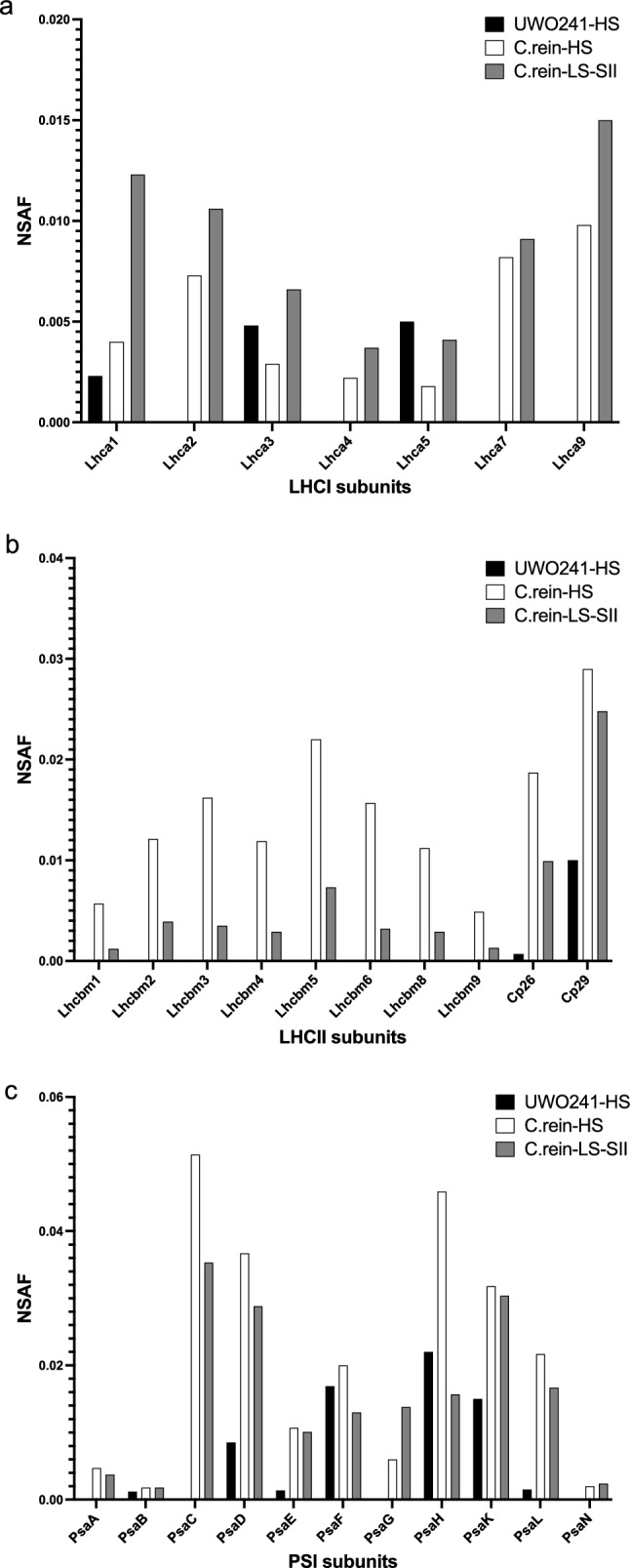
Proteome comparison of light harvesting complexes of supercomplex fractions from *C. reinhardtii* and UWO241. Protein composition of *C. reinhardtii* supercomplexes isolated under state 2 and after high salinity acclimation, as well as UWO241 supercomplex after high salinity acclimation are shown. The normalized spectral abundance factor (NSAF) for each identified protein within a supercomplex was calculated to compare the relative abundance of subunits across species and treatments. The major light harvesting complexes as well as PSI and their subunits participating in supercomplex formation are shown here: Light Harvesting complex I (LHCI, **a**), Light Harvesting complex II (LHCII, **b**) and Photosystem I (PSI, **c**). UWO241-HS (UWO241-HS, Black), *C. reinhardtii*-HS (*C. rein*-HS, White), *C. reinhardtii* state 2 (*C. rein*-SII, Gray)

Compared with supercomplexes described in *C. reinhardtii*, the protein composition of UWO241 PSI-supercomplex exhibits some distinctive features, including the presence of novel phospho-proteins (Szyszka-Mroz et al. 2015) and a depletion in LHCI and LHCII polypeptides (Kalra et al. 2020). We compared PSI-supercomplex composition in *C. reinhardtii* and UWO241 from HS conditions with LS-grown *C. reinhardtii* from state 2 conditions (SII), using shotgun proteomics (Fig. 7, Table 2, Table S1). PSI-supercomplexes from all three samples had proteins of PSI core, LHCI, LHCII, and Cyt b_6_f (Table 2). However, a relatively higher number of polypeptides belonging to these three thylakoid complexes were identified in the supercomplexes from *C. reinhardtii* compared to UWO241. For example, we identified all PSI subunits in *C. reinhardtii* supercomplexes while UWO241-HS supercomplex was missing some, including PsaA, PsaC, and PsaN; although western blotting confirmed the presence of PsaA in the supercomplex and PSI–LHCI bands of UWO241 band (Band 3 and 4, respectively, Fig. S4). In addition, both *C. reinhardtii* supercomplexes contained many subunits of cyt b_6_f, whereas only core subunits PetA, PetB, and PetC as well as PetO were detected in UWO241. This lack of identification of some protein subunits of major complexes in UWO241 supercomplex proteome might reflect low sensitivity of MS data to UWO241 peptides due to absence of a robust database.

**Table 2:** Major proteins involved in supercomplex formation in *C. reinhardtii* and UWO241

Protein complex	UWO241-HS	*C. reinhardtii*-HS	*C. reinhardtii*-State 2
PSI	PsaB, PsaD, PsaF, PsaE, PsaK, PsaH, PsaN	PsaA, PsaB, PsaC, PsaD, PsaE, PsaF, PsaG, PsaK, PsaL, PsaN	PsaA, PsaB, PsaC, PsaD, PsaE, PsaF, PsaG, PsaK, PsaL, PsaN
LHCI	Lhca1, Lhca3, Lhca5	Lhca1, Lhca2, lhca3, Lhca4, lhca5, Lhca7, Lhca9	Lhca1, Lhca2, Lhca3, Lhca4, Lhca5, Lhca7, Lhca9
LHCII	Type I LHCII, Cp26, Cp29	Lhcbm1, Lhcbm2, Lhcbm3, lhcbm4, Lhcbm5, Lhcbm6, Lhcbm8, Lhcbm9, CP26, CP29, Lhcsr3	Lhcbm1, Lhcbm2, Lhcbm3, Lhcbm4, Lhcbm5, Lhcbm6, Lhcbm8, Lhcbm9, CP26, CP29
Cyt b_6_f	Pet A, PetB, PetC, PetO	PetA, PetB, PetC, PetD, PetM, PetO	PetA, PetB, PetC, PetD, PetM, PetO
FNR	*	FNR1	FNR1
FtsH	FtsH2, FtsH 5	FtsH1, 2	FtsH1, 2, 4
OEEP	PsbP, PsbQ	PsbQ, PsbP1	PsbQ, PsbO, PsbP1
Calcium-sensing receptor	CAS	CAS	*

The subunits of each protein identified through shotgun proteomics are shown

*: no protein subunit detected; HS high salinity acclimated; *State 2* state 2 locked culture; *PSI* photosystem I; *LHCI* light harvesting complex I; *LHCII* light harvesting complex II, *Cyt b*_*6*_*f* cytochrome b_6_f, *FNR* ferredoxin NADP reductase, *FtsH* ATP dependent zinc metalloprotease, *OEEP* oxygen evolving enhancer protein, *CAS* calcium-sensing protein

Relative abundance of light harvesting proteins (LHCI and LHCII) and PSI were compared using normalized spectral abundance factor (NSAF) (Fig. 7a–c). Notably, the UWO241 supercomplex was depleted in many LHCII and LHCI proteins compared to PSI supercomplexes from *C. reinhardtii*. LHCI subunit abundance was variable among the different supercomplexes. While *C. reinhardtii*-SII and -HS supercomplexes contained all but one Lhca subunit, we only identified 3 Lhca subunits from PSI-supercomplexes of UWO241 (Fig. 7a). In addition, *C. reinhardtii* PSI-supercomplexes contained the all major LHCII subunits while we could only detect some type I LHCII subunits, cp 26 and cp 29 in the UWO241 PSI-supercomplex (Fig. 7b). Last, HS supercomplex of *C. reinhardtii* had higher NSAF values for most PSI subunits compared to *C. reinhardtii*-SII and UWO241-HS supercomplexes (Fig. 7c).

## Discussion

While CEF plays an important role in plants and algae, balancing ATP/NADPH for carbon fixation (Burlacot et al. 2022; Kramer and Evans 2011; Lucker and Kramer 2013) and photoprotection in both PSII (Joliot and Johnson 2011; Kukuczka et al. 2014) and PSI (Huang et al. 2013, 2017; Yamori and Shikanai 2016), it has been generally associated with response to short-term, transient stress. Whether CEF plays a larger role during either long-term stress acclimation or adaptation to permanent stress (in case of extremophiles) is largely under-studied. The psychrophilic halophyte UWO241 maintains sustained high rates of CEF even though it is a natural state transition mutant (Morgan-Kiss et al. 2002b; Kalra et al. 2020). We wondered whether this phenomenon is an oddity of one extremophilic strain or could high CEF represent a survival strategy to long-term stress in other algae? We exploited *Chlamydomonas* spp. exhibiting a gradient of salt tolerance to delineate the roles of CEF and PSI-supercomplex formation during transient, long-term or permanent stress conditions: (i) *C. reinhardtii*, possessing minimal salt tolerance, (ii) UWO241, exhibiting robust growth and photosynthesis under high salt, (iii) ICE-MDV, showing moderate halotolerance relative to *C. reinhardtii* and UWO241. We attribute the differential salinity sensitivities between the UWO241 and ICE-MDV to the permanent halocline of Lake Bonney: UWO241 lives in the hypersaline lower photic zone, while ICE-MDV occupies the brackish surface waters (Priscu and Spigel 1996; Neale and Priscu 1995).

CEF was activated across the three *Chlamydomonas* spp. strain under variable growth conditions. First, in the absence of salt stress, psychrophilic UWO241 and ICE-MDV exhibited faster CEF rates compared with *C. reinhardtii*. Sustained CEF has been recently reported in other extremophilic algae living in high latitude environments, such as the snow alga *Chlamydomonas nivalis* (Young and Schmidt 2020; Zheng et al. 2020). Thus, adaptation to permanent low temperatures appears to confer constitutively high CEF. Second, all three strains responded to long-term salinity stress by increasing CEF, with the extremophiles exhibiting the fastest CEF rates. In a recent paper we also showed that UWO241 increases CEF in response to long-term high light or low temperatures (Stahl-Rommel et al. 2021). Thus, high CEF is a strategy employed by temperate or extremophilic *Chlamydomonas* to survive either long-term environmental stress or a native habitat of extreme conditions, respectively.

One of the major roles for CEF is additional ATP. In UWO241 CEF rates increased in parallel with increased total proton flux through ATPase and higher ATP production (Kalra et al. 2020). Similarly, CEF activation in HS-acclimated cells of *C. reinhardtii* correlated with increased total proton flux through ATP synthase (Fig. 2c, d). We thus propose that increased CEF can contribute to excess ATP production during long-term salinity stress.

Long-term acclimation or adaptation to high salt is associated with strain-specific remodeling of PSI. UWO241 exhibits downregulated PSI fluorescence emission (at 77 K) across a broad range of treatments and growth conditions (Cook et al. 2019; Morgan et al. 1998), which is associated with reduced levels or loss of LHCI polypeptides (Morgan et al. 1998; Kalra et al. 2020). A recent report also showed that ICE-MDV and *C. reinhardtii* modulated PSI fluorescence emission in response to iron availability, while UWO241 exhibited minimal changes in PSI (Cook et al. 2019). In agreement with Cook et al. (2019), ICE-MDV exhibited PSI functional characteristics that more closely match *C. reinhardtii* (Fig. 3a–c). Thus, under low-salinity conditions, PSI structure appears to be distinct between the Lake Bonney algae, UWO241 and ICE-MDV. This would be an advantage for ICE-MDV which resides in the more variable, less extreme habitat of the near surface of Lake Bonney (Li et al. 2016). Acclimation to high salt induced a loss of PSI fluorescence in *C. reinhardtii* and ICE-MDV, suggesting a common theme of salt-induced PSI remodeling in psychrophilic and mesophilic *Chlamydomonas*.

Sustained CEF is associated with formation of a PSI-supercomplex. A high molecular weight band that migrated lower than PSI–LHCI bands was detected in the sucrose density gradients from high-salt-acclimated cultures of all three of the *Chlamydomonas* species (Fig. 6b). The PSI–LHCI–LHCII supercomplex was first reported in *C. reinhardtii* during state 2 transition and LHCII subunits were shown to be phosphorylated (Takahashi et al. 2006). The state 2 supercomplex was further shown to be calcium-regulated, containing additional CAS, ANR1 and PGRL1 proteins (Terashima et al. 2012). Recently, Steinbeck et al. (2018) provided structural evidence for two structurally distinct supercomplexes in *C. reinhardtii*, a PSI–LHCI–LHCII and PSI–LHCI–Cyt *b*_*6*_*f*. The PSI–LHCI–Cyt b_6_f supercomplex lacked two LHCI proteins (Lhca 2 and 9), while the PSI–LHCI–LHCII supercomplex possessed two LHCII trimers and the full complement of LHCI subunits (Huang et al. 2021). Steinbeck et al. predicted that these structurally distinct supercomplexes have implications on important thylakoid processes: they attributed CEF to the PSI–LHCI–Cyt b_6_f while PSI–LHCI–LHCII was proposed to be more important for rebalancing absorbed energy. Our study also supports the premise that acclimation to short- and long-term stress is associated with functionally distinct supercomplexes. In the *C. reinhardtii*-State 2 supercomplex, both PSI and the supercomplex bands exhibited distinct 77 K fluorescence emission bands at 720 nm, indicative of PSI fluorescence. In contrast, PSI and supercomplex bands collected from C. *reinhardtii*-HS cells exhibited minimal PSI fluorescence and resembled the emission spectra of UWO241 (Fig. 6d, e, g), indicating the absence of LHCI in the supercomplex.

PSI-supercomplexes are ubiquitous; however, protein composition is strain-specific and dependent upon the time scale of stress exposure. Under transient conditions, several proteins were identified in the *C. reinhardtii* PSI-supercomplexes (Iwai et al. 2010; Steinbeck et al. 2018; Takahashi et al. 2006; Terashima et al. 2012). Protein composition of supercomplexes operating under longer term time scales is not known. To further understand the strain- and treatment-specific differences between the supercomplexes, we analyzed the supercomplex components using mass spectrometry. In agreement with previous studies (Huang et al. 2021; Iwai et al. 2010; Steinbeck et al. 2018; Terashima et al. 2012), state 2 (SII) *C. reinhardtii* supercomplexes contained 11 out of 13 PSI subunits (Fig. 7c, Table 2). Similarly, the HS supercomplex of *C. reinhardtii* also contained 11 PSI subunits. On the other hand, we only detected 7 PSI subunits in the UWO241 supercomplex proteome, however major PsaA subunit was identified by western blot (Figs. 7c, S4).

The three supercomplexes also contained varying levels of Lhca proteins. We identified 7 LHCI subunits in both *C. reinhardtii* supercomplexes, however relative to the SII supercomplex, the HS supercomplex of *C. reinhardtii* showed lower NSAF values for all Lhca subunits identified as well as lower LHCI:PSI ratios*.* Thus, reduction in Lhca proteins and a reduced PSI–LHCI peak in the supercomplex 77 K fluorescence emission spectra appear to be part of salinity acclimation in *C. reinhardtii*. In addition to LHCI, LHCII proteins are also an important component of state 2 supercomplexes in *C. reinhardtii*. In agreement with previous reports, we found several lhcb proteins in our SII-supercomplex and more importantly these proteins were also found in HS supercomplex of *C. reinhardtii*. On, the other hand, we could only identify Cp26, Cp29 and type I LHCII subunit in UWO241-HS supercomplex (Table 2). Cyt b_6_f has been shown to be an important member of the state 2 supercomplex, where electron transfer activity revealed reduction of cyt b subunit through ferredoxin (Minagawa 2016). In our study both HS supercomplexes of UWO241 and *C. reinhardtii* contained several cyt b_6_f subunits, including PetO, which has been shown to be important regulator of CEF in *C. reinhardtii* under anoxia (state 2) (Takahashi et al. 2016).

State transitions represent a widely distributed short-term acclimatory mechanism to re-balance excitation energy under conditions of an over-reduced PETC (Wollman 2001). Morgan-Kiss et al. (2002b) proposed that as a consequence to adaptation under permanent stress, UWO241 is ‘locked’ in State 1: a recent paper proposed that UWO241 compensates for a lack of state transitions by a poorly understood energy spill-over mechanism (Szyszka-Mroz et al. 2019). Takizawa et al. (2009) also observed that LS-grown cells of UWO241 were sensitive to oxidizing or reducing conditions of DCMU (state 1) and FCCP (state 2), respectively, while HS-grown cultures remained locked in State 1. While ICE-MDV resides in the same Antarctic lake, it exhibited a typical capacity for state transitions which was comparable with that of *C. reinhardtii* (Fig. 3b). Thus, a loss of state transitions is not a consequence of psychrophily. Instead, when ICE-MDV and *C. reinhardtii* were acclimated to high salinity, both strains exhibited significant attenuation in state transition capacity compared to control conditions (Fig. 3d, e). This phenomenon is further exacerbated in UWO241, owing to adaptation to a hypersaline environment. Furthermore, CEF rates in *C. reinhardtii* under state 1 and state 2 after acclimation to high salinity showed minimal difference and displayed constitutive upregulation in both conditions compared to state 1 condition in low salinity (Fig. S5). Therefore, it appears that non-stressed cells are primed to respond to short-term perturbations with state transitions, while long-term acclimation or adaptation to salt causes structural changes to PSI-supercomplexes which prevent this response via constitutive upregulation of CEF.

Adaptation to low temperatures and high salinity is associated with differential thylakoid phospho-protein patterns. The aberrant capacity for state transitions in UWO241 was previously linked with an inability to phosphorylate major LHCII polypeptides (Morgan-Kiss et al. 2002b). Instead, novel, high molecular mass phospho-proteins of > 130 kD as well as a 17 kD polypeptide identified as a PsbP-like protein were identified in the thylakoids and supercomplexes isolated from HS-grown UWO241 cells (Szyszka-Mroz et al. 2015). More recently, Szyszka-Mroz et al. (2019) reported that UWO241 does exhibit light-dependent [γ-33P] ATP labeling of thylakoid polypeptides, including limited phosphorylation of LHCII proteins. The phospho-protein patterns were unique in UWO241 compared with *C. reinhardtii*, and a cold-active kinase was identified in UWO241 (Szyszka-Mroz et al. 2019). In the current study, we confirmed the unique phosphorylation patterns of UWO241 thylakoids relative to *C. reinhardtii*, with minimal phosphorylation of LHCII and the appearance of multiple high molecular weight phospho-proteins. In contrast, phospho-protein patterns in ICE-MDV exhibit features of both UWO241 and *C. reinhardtii*, with the presence of major LHCII phospho-proteins and the appearance of higher molecular weight bands (Fig. 5). These differences between UWO241 and ICE-MDV fit well with the retainment of state transition ability in ICE-MDV.

In contrast with our findings that acclimation and adaptation to salinity stress interferes with state transition capacity in *Chlamydomonas* species, the model halophile *Dunaliella* is capable of state transitions (Li et al. 2019; Petrou et al. 2008). State transition ability in *Dunaliella* appears to be associated with different configurations of PSI. Perez-Boerema et al. (2020) isolated a minimal PSI from *D. salina* which is missing several PSI core subunits which are necessary for state transitions (Perez-Boerema et al. 2020). The structure of the ‘Basic PSI’ had only 7 core subunits (PsaA-F; PsaJ). Caspy and colleagues then isolated a ‘Large PSI’ containing additional core subunits, including PsaL and PsaO (Caspy et al. 2020). The authors suggest that small and large PSI conformations allow green algae to modulate the function of PSI in variable environments. Our findings extend these recent structural studies by linking PSI-supercomplexes with PSI function. The salt-tolerant UWO241 supercomplex appears to possess the small conformation of PSI, while the salt-sensitive *C. reinhardtii* supercomplex appears to contain the larger PSI. Further research is needed to better understand the stability and functional differences between the green algal PSI conformations.

**Fig. 8:**
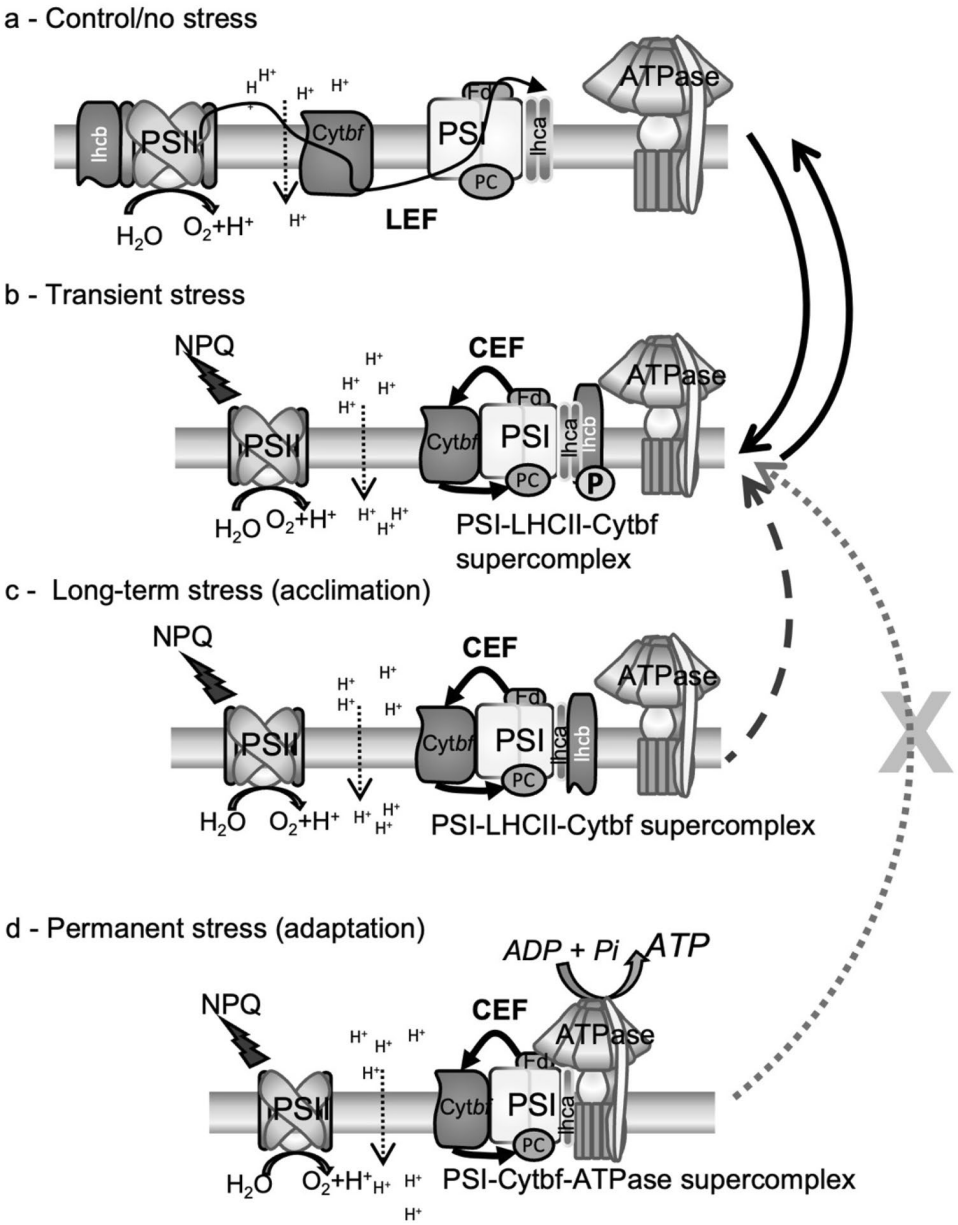
Proposed model for formation of CEF-associated PSI-supercomplexes during short- and long-term stress in *Chlamydomonas* spp. **a** Structure of the photosynthetic electron transport chain (PETC) during optimal growth condition. Note that linear electron flow (LEF) constitutes the bulk of electron transport during this condition and major protein complexes are not physically associated. **b** PETC under conditions of state 2 after incubation in short-term stress condition (Transient stress). Note that PSI is associated with LHCI, cytochrome b_6_f and LHCII proteins to form a PSI-supercomplex that initiates increased CEF around PSI, and excess energy is quenched through NPQ response via acidification of lumen side. Solid arrows indicate the bi-directionality of capacity for the organism to go from transient state (state 2) back to control state (state 1) via dissociation of major protein complexes. **c** PETC configuration after long-term acclimation to salinity stress. Note that PSI is associated with cytb_6_f, LHCI (reduced) and LHCII proteins to form a stable PSI-supercomplex that initiates CEF around PSI and increased total proton flux leads to increased ATP synthesis and quenching of excess energy by NPQ. Dotted arrow shows decrease in capacity for state transition after acclimation to long-term salinity stress due to the presence of stable PSI-supercomplex. **d** PETC configuration after adaptation to permanent salinity stress, as in case for halotolerant psychrophile UWO241. Note that PSI is associated with cytb_6_f, LHCI (reduced), LHCII proteins as well as ATP synthase to induce constitutive CEF around PSI and increased total proton flux leads to increased ATP synthesis and quenching of excess energy by NPQ. Here the dotted arrow with cross symbolizes the complete loss of state transition potential due to presence of stable PSI-supercomplex and constitutively upregulated CEF. For simplicity, other small proteins that are part of PSI-supercomplex are not shown. Model is based on results from this study and previous work (Morgan-Kiss et al. 2002b; Kalra et al. 2020).

We present an updated model to delineate between the effects of transient stress vs long-term stress vs adaptation on the photosynthetic apparatus of *Chlamydomonas* species (Fig. 8). Under control/no stress state, the major protein complexes of the PETC are not attached, and the majority of the electron transport takes place as LEF, producing ATP and NADPH (Fig. 8a). Based on previous studies, during a transient/short-term stress state, the organism forms a transient PSI–LHCI–LHCII–Cytb_6_f supercomplex to initiate CEF around PSI, increasing transthylakoid pH and inducing NPQ response to balance excess energy (Iwai et al. 2010; Steinbeck et al. 2018) (Fig. 8b). Dissociation of this supercomplex can occur as the transient stress dissipates (bidirectional arrows). In contrast with the transient response to stress, both salt-sensitive *C. reinhardtii* and moderately salt-tolerant ICE-MDV attenuated their state transition capacity but also constitutively upregulated CEF after acclimation to long-term salinity stress. So, what structural or functional alterations in the PETC associated with long-term salinity acclimation could be contributing to altered state transition response but constitutively high CEF? A major consequence of a state transitions is formation of PSI–LHCI–LHCII–Cytb_6_f supercomplex and consequent increase in CEF (Iwai et al. 2010) (Fig. 8b). However, Takahashi et al. (2013) elucidated that although both CEF and state transitions are controlled through redox status of the plastoquinone pool, they can occur independent of each other. Based on the results of this study, during long-term stress acclimation, the organism can form a stable supercomplex composed of PSI, LHCI (reduced LHCI:PSI compared to state 2, Fig. 7), LHCII, cyt b_6_f, and other minor proteins to initiate constitutive CEF around PSI that may produce ATP as well as induce NPQ response (Fig. 8c) independent of state transitions. We propose that this restructuring of the PETC to provide sustained CEF through a stable PSI-supercomplex inhibits the classic state transition response in *Chlamydomonas* species (Fig. 8, dotted arrow). Last, we describe the PETC confirmation during adaptation to permanent stress. As we have previously shown in salinity-adapted UWO241, a restructured photosynthetic apparatus with a stable supercomplex composed of PSI, LHCI (reduced), cytb_6_f, ATP synthase, and other minor proteins, is primed for constitutive capacity for CEF and is correlated with excess ATP production and increased NPQ capacity (Kalra et al. 2020, Fig. 8d). Concomitantly, UWO241 has completely lost the capacity for classic state transition (Morgan-Kiss et al. 2002b) and appears to rely on a poorly understood spill-over mechanism for titering absorbed energy between the photosystems (Szyszka-Mroz et al. 2019). We, thus, propose that assembly of stable PSI supercomplexes could be a general acclimatory response to deal with long-term high salinity in *Chlamydomonas* species.

## Conclusions

As the climate change and human activities exacerbates the impact of environmental stressors such as salinization, there is a growing need for an improved understanding of how organisms will respond to and survive a myriad of stress conditions, especially long-term steady-state stresses (Alexandratos and Brunismas 2012). Maintaining optimal function of the photosynthetic apparatus is key for survival under environmental change. CEF is an important pathway in almost all photosynthetic organisms, making it an ideal candidate to study stress acclimation in the context of climate change (Kramer and Evans 2011). Understanding how CEF can help plant and algal survival under physiologically relevant, steady-state stress conditions can help us to engineer photosynthetic organisms to better withstand climate change in the future.

In this study, we show that sustained CEF supported by restructuring of PSI and formation of a supercomplex is an important strategy in *Chlamydomonas* spp. to deal with long-term high salinity stress. CEF has the dual benefit of providing photoprotection of both PSI and PSII and balancing energy needs. Our study suggests that green algae adapted to different environmental stressors have evolved to activate CEF and titer the stability of the PSI-supercomplex to support stress responses over broad time scales.

## Supplementary Information

Below is the link to the electronic supplementary material.

Supplementary file1 (PDF 652 kb)

Supplementary file2 (XLSX 96 kb)—**Table S1** Proteome data for supercomplexes isolated from UWO241-HS, *C. reinhardtii*-HS and *C.reinhardtii*-LS-state2. Detailed information on the identified peptides such as Uniprot identifier, peptide count, protein coverage, protein score, NSAF values and description is included in the table.

## Data Availability

UWO241 transcriptome is available on NCBI database with Accession Number: PRJNA575885.

## Abbreviations

ANR1: Anaerobic response I protein
ATPase: ATP synthase
Cyt b_6_f: Cytochrome b_6_f
CEF: Cyclic electron flow
CAS: Calcium-sensing protein
DIRK: Dark interval relaxation kinetics
ECS: Electrochromic shift
FNR: Ferredoxin NADP reductase
*F*_V_/*F*_M_: Maximum photosynthetic efficiency of photosystem II
HS: High salt
LS: Low salt
LEF: Linear electron flow
LHCI: Light harvesting complex I
LHCII: Light harvesting complex II
NPQ: Non-photochemical quenching
PSI: Photosystem I
PSII: Photosystem II
PETC: Photosynthetic electron transport chain
PGRL1: Proton gradient-like protein 1
SII: State 2
*t*_1/2_^red^: Half-time for P700 re-reduction
