# Supplementary material for: High salt-induced PSI-supercomplex is associated with high CEF and attenuation of state transitions
Source: Photosynth Res. 2023 Jun 22;157(2-3):65–84. doi: 10.1007/s11120-023-01032-y (PMC10484818; doi:10.1007/s11120-023-01032-y)
Supplement: Supplementary file 1 — Supplementary file1 (PDF 652 kb) [file 11120_2023_1032_MOESM1_ESM.pdf]

High salt-induced PSI-supercomplex is associated with high CEF and attenuation of state-transitions

Isha Kalra [a, c], Xin Wang[a], Ru Zhang[b], Rachael Morgan-Kiss[a]

[a] Department of Microbiology, Miami University, Oxford, Ohio 45056, USA

[b] Donald Danforth Plant Science Center, St. Louis, Missouri 63132, USA

[c] Present address: Department of Biology, University of Southern California, Los Angeles, CA 90089, USA

SUPPLEMENTARY MATERIAL

Table of Contents

Fig. S1: Growth rates of the three *Chlamydomonas* species under salinity gradient.

Fig. S2: P700 reduction kinetics of the three *Chlamydomonas* species under low and high salinity.

Fig. S3: PSII state transition test for the three *Chlamydomonas* spp under low (LS) and high (HS) salinity.

Fig. S4: Immunoblot of PsaA in UWO241-HS thylakoids and protein complex fractions collected from sucrose density gradient centrifugation.

Fig. S5. Interacting impacts of high salinity acclimation and state transition capacity on P700 activity in *C. reinhardtii*.

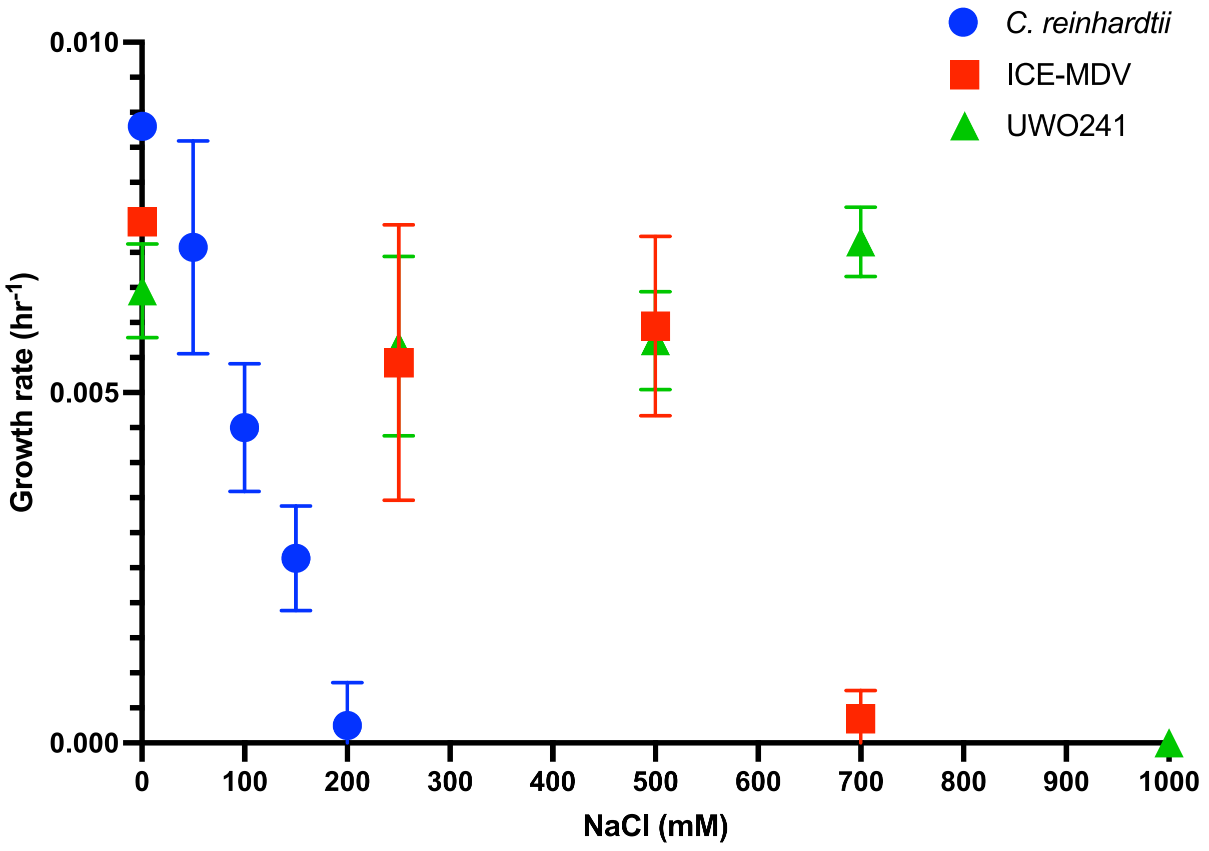

Fig. S1: Growth rates of the three *Chlamydomonas* species under salinity gradient. The model mesophile *C. reinhardtii* (blue, circle) was grown in salinity levels of 0.43 mM, 50 mM, 100mM, 150 mM and 200 mM NaCl. Both psychrophiles, ICE-MDV (red square) and UWO241 (green triangle) were grown in salinity levels of 0.43 mM, 250 mM, 500 mM and 700 mM NaCl, with an additional salinity level of 1000mM NaCl for UWO241. (*n*=3, ±SD). From these growth rates maximum salinity level that a strain can tolerate while maintaining their growth rates were selected for further experiments.

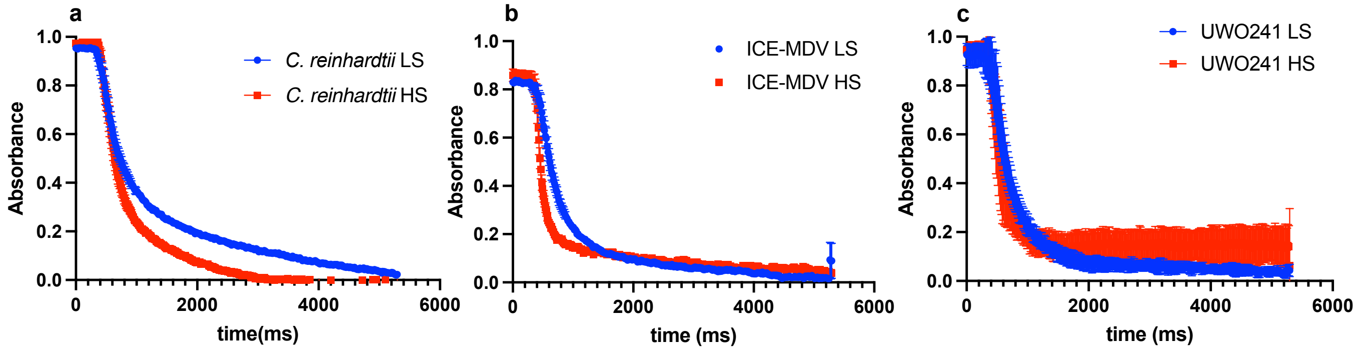

Fig. S2: P700 reduction kinetics of the three *Chlamydomonas* species under low and high salinity. a. *C. reinhradtii* , b. ICE-MDV, c. UWO241. Low salinity (LS) traces are shown in blue. High salinity (HS) traces are shown in red.

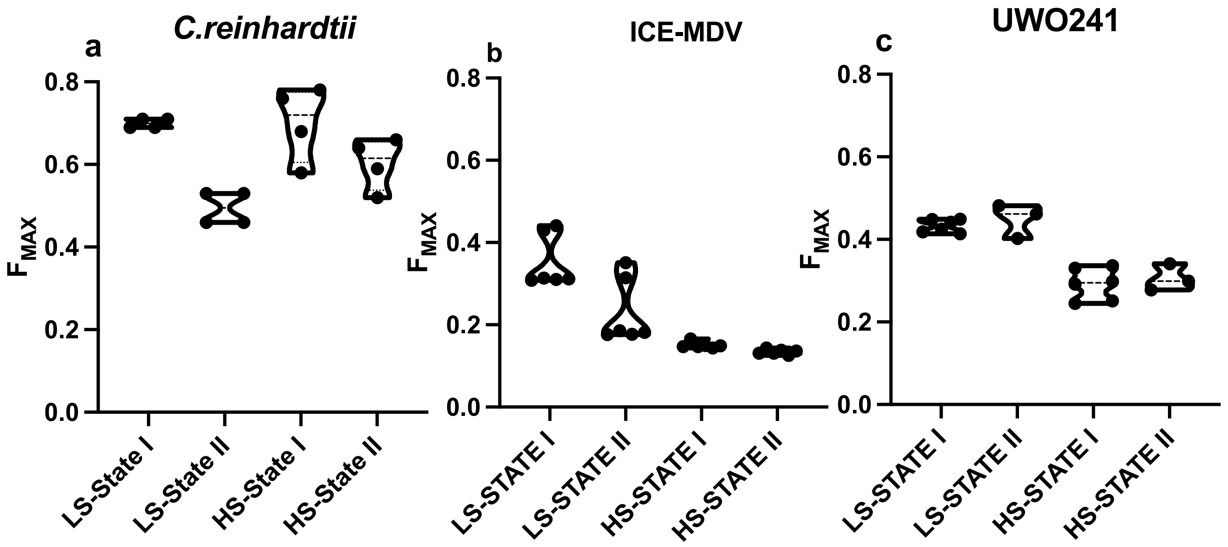

Fig. S3: PSII state transition test for the three *Chlamydomonas* spp under low (LS) and high (HS) salinity. The maximum PSII fluorescence values (F_{MAX}) are shown for all three strains (*C.reinhardtii*-a, ICE-MDV-b and UWO241-c) under state I and state 2 conditions. State I: DCMU, State II: FCCP. (*n*=4-6, dotted line=mean value)

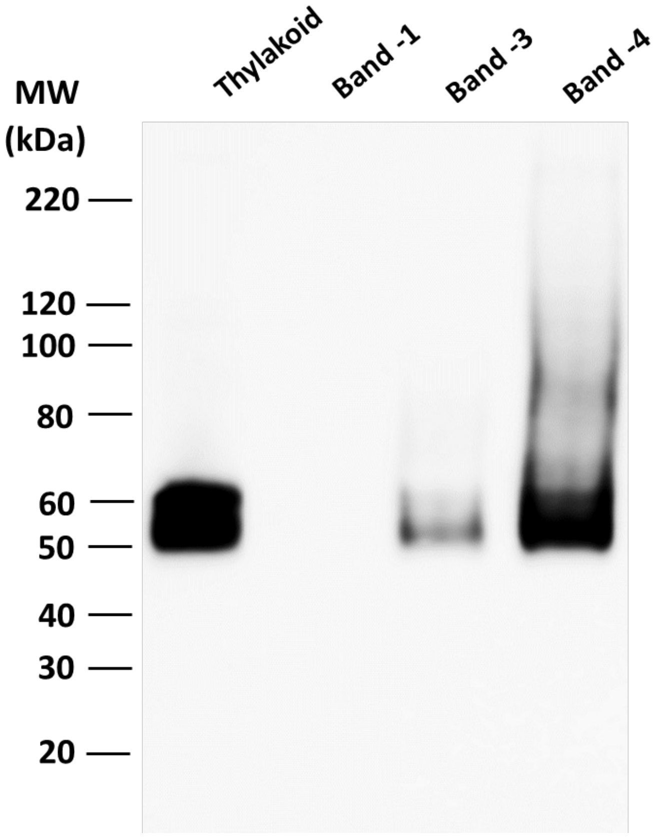

Fig. S4: Immunoblot of PsaA in UWO241-HS thylakoids and protein complex fractions collected from sucrose density gradient centrifugation. Band-1: LHCII complex, Band-3: PSI-LHCI complex, Band-4: Supercomplex. Molecular weight ladder (kDa) is shown on left.

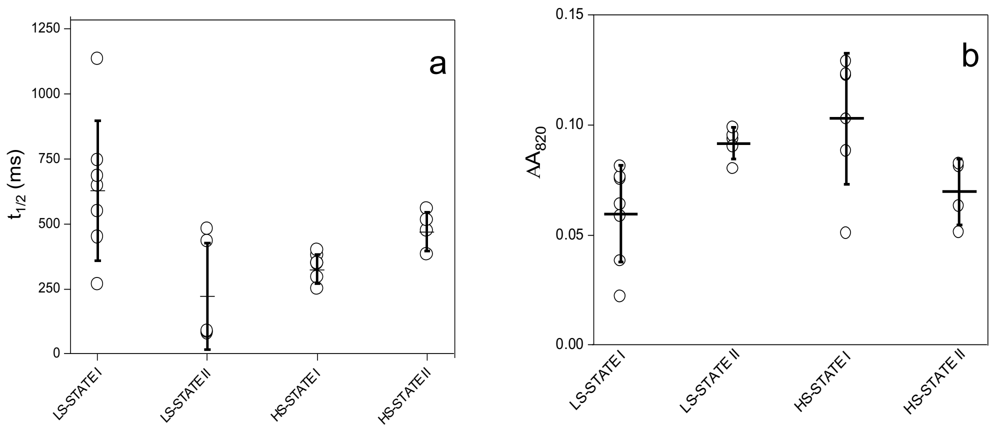

Fig. S5. Interacting impacts of high salinity acclimation and state transition capacity on P700 activity in *C. reinhardtii*. Log-phase cultures acclimated to either low salt (LS) or high salt (HS) were incubated under State I or State II conditions prior to P700 oxidation/reduction measurements. a. CEF rates measured as re-reduction time. b. Change in absorbance of P700. (n=6±SD).
